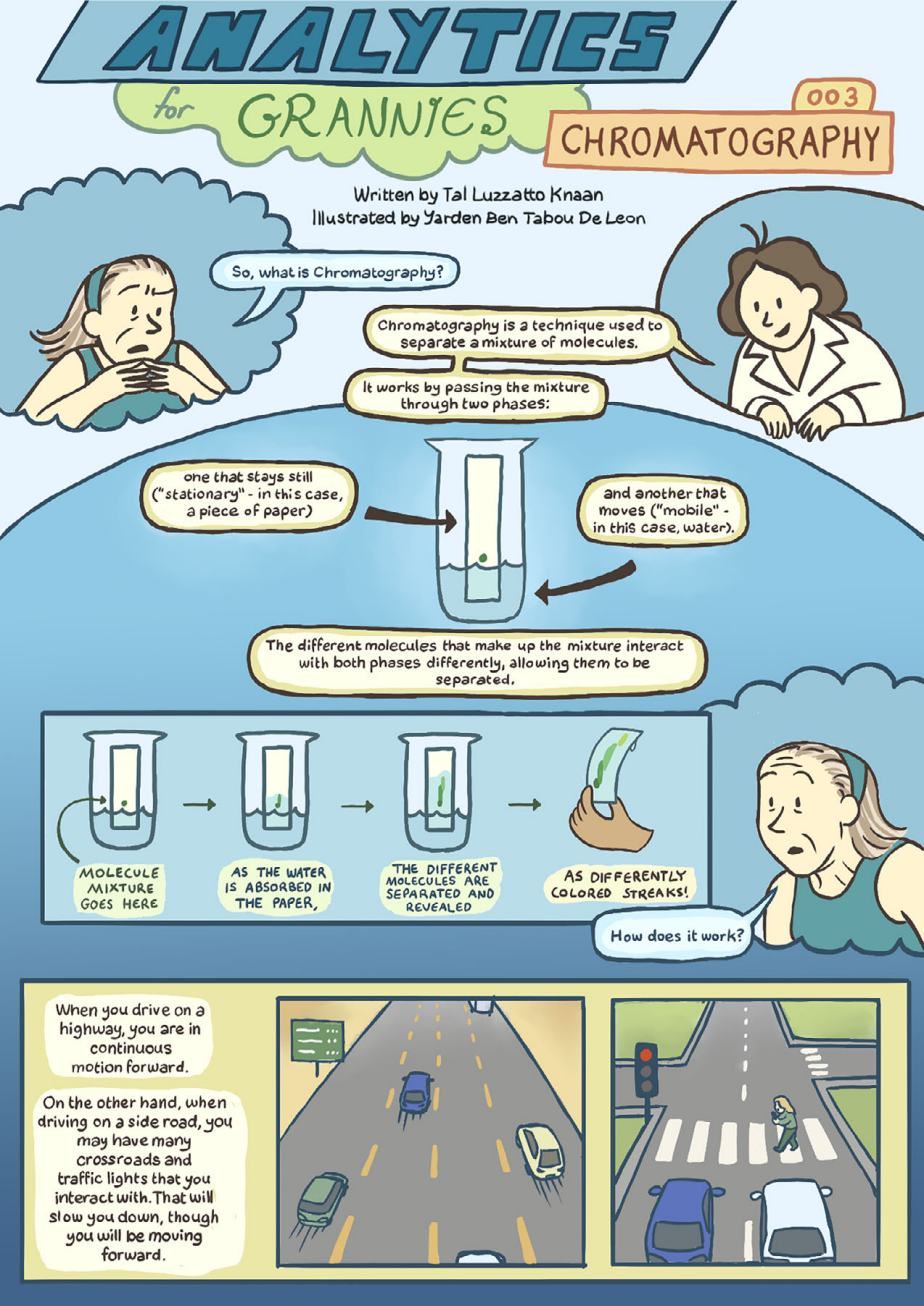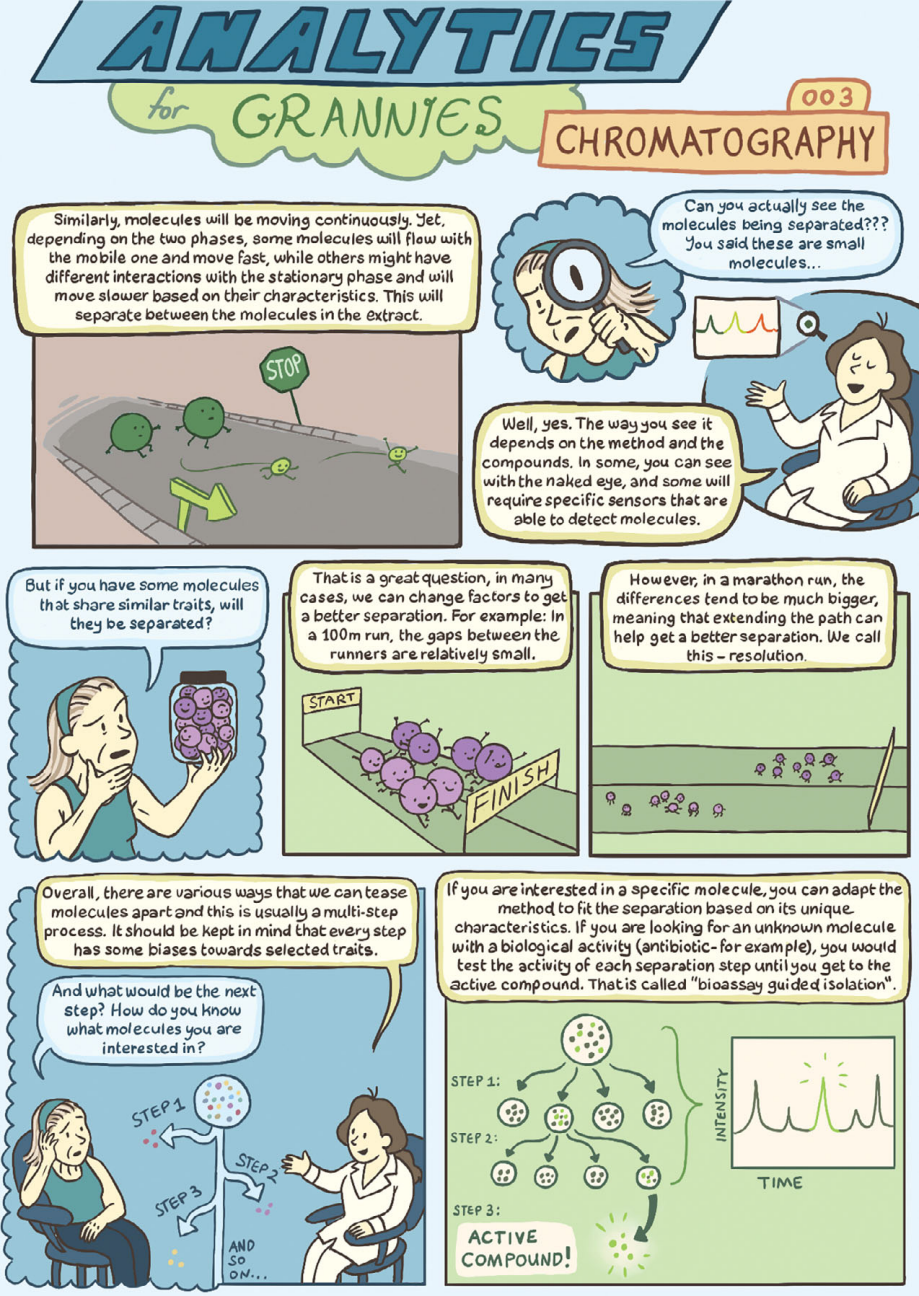# Analytics for Grannies 003: Chromatography

**DOI:** 10.1002/ansa.202400087

**Published:** 2025-01-08

**Authors:** Tal Luzzatto Knaan

**Affiliations:** ^1^ Department of Marine Biology, The Leon H. Charney School of Marine Sciences University of Haifa, Mount Carmel Haifa Israel